# The effect of long-term e-cigarette use on the inflammation marker fractional exhaled nitric oxide (FeNO): A systematic review and meta-analysis

**DOI:** 10.18332/tid/224361

**Published:** 2026-09-30

**Authors:** Mohammed A. Almeshari, Afaf M. Alrashdi, Alhanouf G. Almutairi, Lujain A. Alfaiz, Rimas M. Almalki, Salma A. Kaabi

**Affiliations:** 1 Institute of Inflammation and Ageing, University of Birmingham, Birmingham, United Kingdom; 2 Rehabilitation Health Sciences Department, College of Applied Medical Sciences, King Saud University, Riyadh, Saudi Arabia; 3 Rehabilitation Sciences Department, Faculty of Health and Rehabilitation Sciences, Princess Nourah Bint Abdulrahman University, Riyadh, Saudi Arabia

**Keywords:** e-cigarettes, conventional cigarettes, inflammation, FeNO

## Abstract

**INTRODUCTION:**

E-cigarettes are perceived as a less harmful alternative to conventional cigarettes; however, concerns remain about the potential health risks associated with long-term use. This systematic review and meta-analysis aimed to assess the long-term effects of e-cigarettes on airway inflammation, as measured by fractional exhaled nitric oxide (FeNO).

**METHODS:**

This is a systematic review and meta-analysis. MEDLINE and EMBASE were searched via Ovid, with the last search conducted on 3 October 2025. Studies were included if they used FeNO to assess the effects of long-term e-cigarette use; those without FeNO measurements were excluded. Risk of bias was assessed with validated tools according to study design, and meta-analysis was performed when quantitative data were available. The protocol was prospectively registered in PROSPERO (CRD42024508780).

**RESULTS:**

From 1018 records, six studies (n=792 participants) met the eligibility criteria. The included studies varied in population characteristics and findings: one reported higher FeNO in complete quitters versus failures/reducers; two found higher FeNO in e-cigarette users versus controls; two observed no difference; and one found lower FeNO among e-cigarette users. The meta-analysis of three studies showed a pooled mean difference of -0.26 (95% CI: -9.74–9.22), indicating no significant difference between groups.

**CONCLUSIONS:**

The results across studies were heterogeneous and inconclusive, highlighting the variability in findings related to the impact of long-term e-cigarette use on airway inflammation. These inconsistencies underscore the need for further research, particularly well-powered longitudinal prospective studies, to better understand the long-term effects of e-cigarette use on inflammatory biomarkers such as FeNO.

## INTRODUCTION

E-cigarettes affect the respiratory system, altering airways and lung development, and influencing nitric oxide (NO) levels^1^. NO, produced by respiratory tract cells, plays a role in airway function and diseases. Elevated exhaled NO (eNO) is associated with higher eosinophil counts, particularly in asthma, allergic rhinitis, and COPD^2,3^. Fractional exhaled nitric oxide (FeNO) is an easy and affordable test to measure nitric oxide (NO) levels, which can indicate inflammation^2,4^. High FeNO levels suggest airway inflammation, while smoking typically reduces FeNO by inhibiting nitric oxide synthase (NOS)^1,3^. Research gaps remain regarding e-cigarettes’ effects on inflammation, with most studies focusing on smoke exposure biomarkers. Studies on long-term e-cigarette use, such as murine models, show similarities to human COPD, including increased pro-inflammatory cytokines^5^. Smoking-related inflammation involves increased macrophages, lymphocytes, and other immune cells in the lungs^6^. FeNO reduction in smokers is linked to neutrophilic inflammation^5^. Protease concentrations, another potential biomarker, are also elevated in smokers^5^.

Given the lack of evidence on the long-term effects of e-cigarettes, this study specifically focuses on whether e-cigarette use for more than three months is associated with inflammatory dysfunction, as assessed by fractional exhaled nitric oxide (FeNO).

## METHODS

This is a systematic review and meta-analysis, with its protocol registered in the International Registry of Systematic Reviews (PROSPERO) with the number CRD42024508780. The reporting items for Systematic Reviews and Meta-Analyses (PRISMA) guidelines were used in reporting the review^7^, and the checklist is provided in the Supplementary file Appendix 1. The FeNO test was chosen due to its ability to identify possible airway inflammation.

### Searching strategy

Details of the search strategy are provided in Supplementary file Appendix 2, and search terms covering subject headings and keywords related to FeNO and e-cigarettes are listed in Supplementary file Appendix 3. Searches were performed using the EMBASE and MEDLINE databases. The literature search was conducted in February 2024, with the most recent search on 3 October 2025.

The population was adults aged ≥18 years; intervention/exposure was use of electronic cigarettes (e-cigarettes or vaping devices); comparator was individuals with no e-cigarette exposure, including non-smokers or conventional cigarette smokers when applicable; and outcome was fractional exhaled nitric oxide (FeNO) levels measured as an indicator of airway inflammation.

### Eligibility criteria

Studies were eligible for inclusion if they assessed the effects of long-term e-cigarette use (≥3 months) on airway inflammation in healthy adults (≥18 years) using the fractional exhaled nitric oxide (FeNO) test. Eligible studies also included comparisons with control groups (individuals not exposed to e-cigarettes) and referenced established FeNO ranges. Studies were excluded if they focused exclusively on short-term e-cigarette use (<3 months) or individuals with pre-existing respiratory diseases. No restrictions were placed on the study design, provided primary data were reported.

### Study selection

The search results were imported into EndNote 9.1 by Clarivate Analytics where duplicates were removed automatically, and then data were uploaded to Rayyan, a web-based application tool used for screening abstracts^8^. MAA, AGA, and AMA independently and blindly screened the abstracts using preset inclusion and exclusion criteria. Disagreements that arose were solved through discussion. MAA loaded full-text articles into EndNote 9.1 after obtaining them, and the same method was used for screening the full texts for eligibility.

### Data extraction

The authors, AMA and AGA, gathered and confirmed accurate and consistent data using a customized, piloted Excel sheet for data extraction. The extracted data included general information about the study (authors, year of publication, and title); study characteristics (study design, country/location, sample size, exposure duration); participants’ demographics (age range, gender distribution, smoking status, e-cigarette user status, and baseline FeNO levels); exposure details (type of e-cigarette, duration of e-cigarette use, control group description); outcomes (change in FeNO levels, measurement time points, measurement technique/device, FeNO levels, description, and results). Lastly, key findings were extracted from each included study, including a summary of the main results comparing e-cigarette users with control groups, as well as reported confounding factors, participant comorbidities, study limitations, and other relevant notes described by the study authors. Confounding factors that could potentially influence FeNO levels were identified based on information reported in the included studies and considered during the narrative evaluation. These included prior or current use of conventional cigarettes, marijuana vaping, the presence of COPD, asthma, or allergic rhinitis, inclusion of pediatric participants, and short-term e-cigarette use (defined as <3 months).

### Synthesis methods

The study data were synthesized quantitatively when studies were sufficiently homogeneous in design and outcomes. Heterogeneity was assessed using the I^2^ statistic to determine whether pooling of results was appropriate. DerSimonian and Laird’s random-effects model was applied to calculate the overall effect estimates and corresponding 95% confidence intervals (CI), with the inverse variance approach used to compare the mean difference (calculated as mean FeNO in the e-cigarette group minus the mean FeNO in the control group). Results of the quantitative synthesis were presented using forest plots and summary tables. When pooling was not feasible due to heterogeneity of outcomes or reporting, a qualitative narrative synthesis was performed. A descriptive analysis of study characteristics (e.g. design, setting, number of participants, analysis methods), methodological quality, and reported estimates was also undertaken. Where necessary, data were standardized to consistent units to enable comparability across studies. The Meta-analysis of Observational Studies in Epidemiology (MOOSE) standards were followed in reporting the observational studies, and the checklist is provided in the Supplementary file Appendix 4.

### Quality and risk of bias assessment

The quality of cohort and cross-sectional studies was assessed using the validated National Institutes of Health (NIH) Quality Assessment Tools, with studies classified as poor, fair, or good^9^. Two reviewers independently performed the quality assessments, and disagreements were resolved through discussion and consensus. In addition, potential reporting bias and publication bias were explored through visual inspection of funnel plots when sufficient studies were available; however, given the small number of included studies, formal statistical testing for publication bias was not feasible.

## RESULTS

### Study selection

In the initial search, a total of 1018 abstracts were identified from the MEDLINE and Embase databases using the relevant keywords. A total of 13 clinical trials were found on the clinicaltrias.gov portal. After the removal of duplicates, out of 976 screened abstracts, 969 were excluded for not meeting the inclusion criteria. Seven articles were included for full-text screening, and one was excluded as a review article. Ultimately, six articles met the inclusion criteria^10-15^. Articles excluded in the full-text screening phase are described in the PRISMA flow diagram, with reasons given and illustrated in Figure 1.

**Figure 1:**
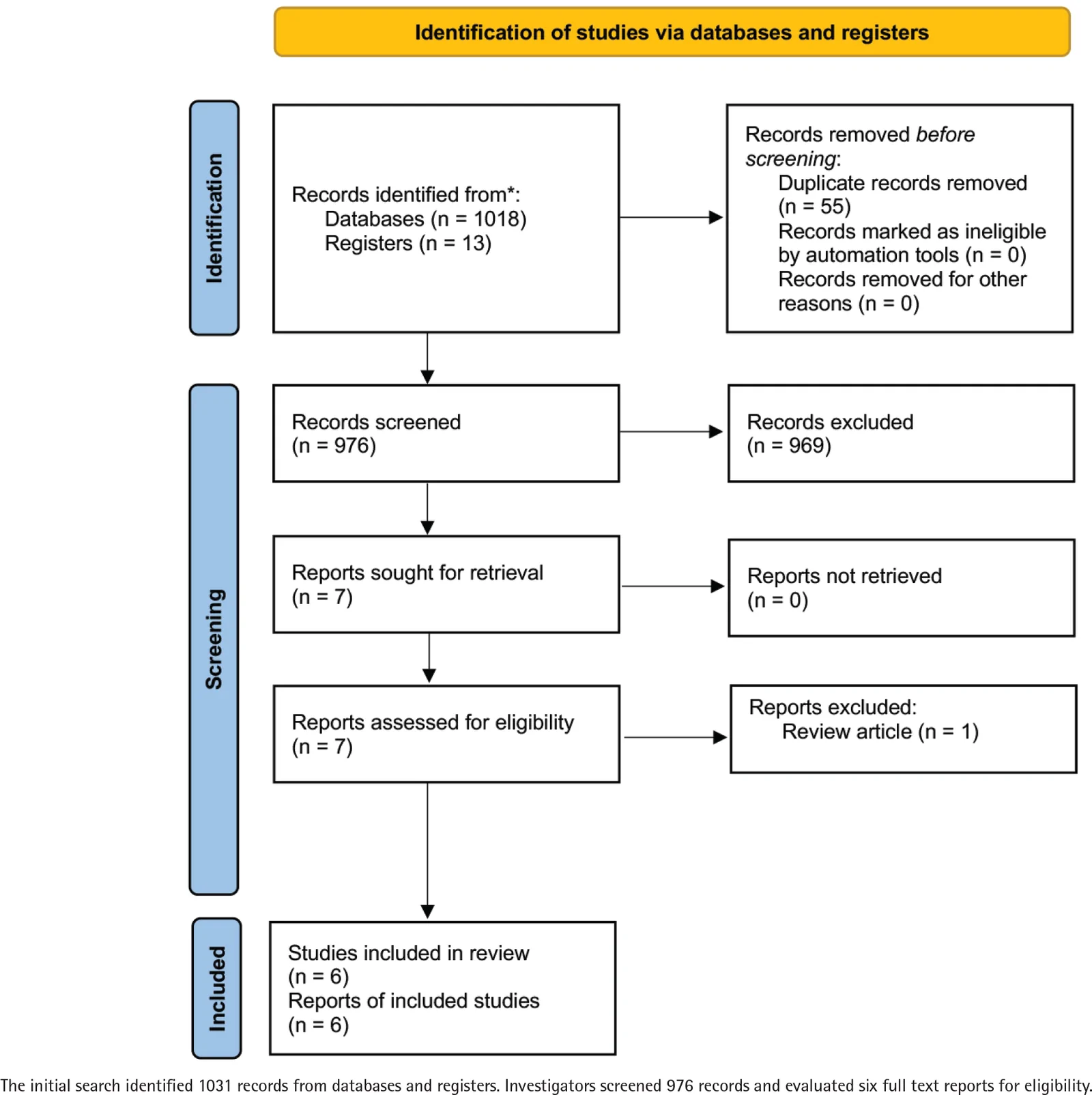
PRISMA flow diagram detailing the literature search and study selection process for the systematic review

### Study characteristics

Six studies, with a total sample size of 792 (397 e-cigarette users, 218 controls, and 177 traditional cigarette smokers), were included^10-15^. Meo et al.^12^ reported e-cigarette users’ FeNO levels as 18 ± 11.05 ppb, which were lower than controls 21.67 ± 8.58 ppb (p=0.16). Tattersall et al.^15^ reported FeNO levels lower in e-cigarette users, 14.5 ± 14.7 ppb, compared to controls, 16.2 ± 11.0 ppb (p=0.013). Polosa et al.^13^ after 3.5 years of usage observed 20.0 ppb in both groups (p=0.89) suggesting no difference. Majek et al.^11^ reported e-cigarette users having 16.9 ± 6.5 ppb of FeNO, which was higher than the controls who had 13.2 ± 5.9 ppb (p=0.01). Campagna et al.^10^ reported that participants who quit smoking via e-cigarette use showed a significant increase in FeNO levels from 5.5 ppb to 17.7 ppb over a 52-week period. Sompa et al.^14^ reported significantly higher FeNO levels in e-cigarette users (median: 14 ppb, p=0.04) compared to healthy non-smokers (11 ppb), while conventional cigarette smokers had lower FeNO levels (9 ppb, p=0.04). Four of the included studies were conducted in different countries (Saudi Arabia, Poland, Sweden, and the United States), and the other two studies were conducted in Italy. The mean age of participants in these studies varied from 18 to 56 years. The largest sample size belonged to one study with 395^15^, while two studies had a sample size ranging from 100 to 160^10,11^, and another three studies had a sample size less than 100^12-14^. The studies had different proportions of males and females in terms of gender distribution: one of the six studies^11^ had an equal distribution between two genders; another one^12^ included only male participants, and the remaining four studies had fewer female than male participants^10,13-15^. Some of the studies differed in the duration of e-cigarettes used, for example, one recruited subjects with e-cigarettes use of more than 12 weeks^10^, another extended for at least 6 months^12^, and the third only followed participants for three and a half years^13^. One study also reported a median duration of 4 years^15^, another study reported a median e-cigarette use duration of 2.7 years^14^, while another study did not specify the duration of e-cigarette use^11^. The participants’ smoking status varied across the studies. In one study, participants were required to smoke at least 10 conventional cigarettes per day for a minimum of five years^10^. Another study focused on individuals who had never used conventional cigarettes^12^. In one study, participants were categorized into four groups based on their smoking habits: conventional cigarette smokers, heated tobacco users, e-cigarette users, and non-smokers^11^. Another study involved three groups: control group, e-cigarette user group, and cigarette smoker group^15^. One study also enlisted those who had never smoked or smoked fewer than 100 cigarettes^13^. Sompa et al.^14^ included four groups: e-cigarette users, conventional cigarette smokers, dual users, and healthy non-smokers. The demographic data of all participants are reported in Table 1.

**Table 1:** Participant demographics and fractional exhaled nitric oxide measurements across all included studies

*Study*	*Design*	*Variable*	*EC users group*	*Control group*		*Traditional cigarette*	*p*
Sompa et al.^14^ 2025	Cross-sectional	Age	27 (24–29)	27 (24–36)		30 (24.5–39.5)	0.3
		Gender (M/F)	10/10	13/9		6/14	NR
		Body mass index	23.7 (20.8–26)	23.9 (22–27.4)		23.2 (21.2–28.3)	0.8
		FeNO (ppb)	Median: 14	Median: 11		Median: 9	0.04
Majek et al.^11^ 2023	Cross-sectional	Age	27.07 ± 6.0	25.90 ± 7.72		23 (21–24.5)	<0.01
		Gender (M/F)	20/20	19/21		18/22	NR
		Body mass index	NR	NR		NR	NR
		FeNO (ppb)	16.9 ± 6.5	13.2 ± 5.9		10.3 ± 5.7	<0.01
Tattersall et al.^15^ 2023	Cross-sectional	Age	42.8 ± 13.8.	30.8 ± 11.9		42.8 ± 13.8	<0.001
		Gender (M/F)	100/64	57/57		65/52	NA
		Body mass index	25.7 ± 5.7	24.8 ± 4.7		28.3 ± 7.5	NA
		FeNO (ppb)	14.5 ± 14.7	16.2 ± 11.0		9.7 ± 12.3	0.013
Polosa et al.^13^ 2017	Cohort	Age	29.7 ± 6.1	32.5 ± 7.0		NR	0.61
		Gender (M/F)	6/3	8/4		NR	NR
		Body mass index	NR	NR		NR	NR
		FeNO* (ppb)	21.1 (16.2–24.5)	18.6 (17.6–25.7)		NR	0.89
Meo et al.^12^ 2018	Cross-sectional	Age	27.07 ± 6.00	25.90 ± 7.72		NR	0.51
		Gender (M/F)	30 males only	30 males only		NR	NR
		Body mass index	28.46 ± 7.29	28.84 ± 5.97		NR	0.82
		FeNO (ppb)	18 ± 11.05	21.67 ± 8.58		NR	0.16
Campagna et al.^10^ 2016	Randomized Controlled Trial (RCT)	Group	Failures	Reducers	Quitters	NR (Pre-post)	
		Age	41.6 ± 13.0	45.4 ± 14.4	44.8		0.28
		Gender (M/F)	43/39	22/12	14/4		0.13
		Body mass index	NR	NR	NR	NR	NR
		FeNO* (ppb)	6.6 (4.3–8.4)	5.9 (5.0–7.9)	5.5 (4.5–6.9)		0.47

The table details baseline characteristics for 730 total participants. The participant groups consist of electronic cigarette users, control subjects, and traditional cigarette smokers. The documented clinical variables include age, gender, body mass index, and fractional exhaled nitric oxide levels. Data presentation includes mean with standard deviation (mean ± SD) or median (IQR). NR: not reported. M/F: male and female. FeNO: Fractional exhaled Nitric Oxide. EC: electronic cigarette.

### Quality assessment

This systematic review used quality evaluation methods based on the design of the included studies, using the NIH tools for quality assessment. Four studies were cross-sectional studies^11,12,14,15^, one cohort^13^, and one randomized controlled trial (RCT)^10^. The RCT was assessed separately using the NIH quality assessment tool for RCTs, where it was rated as Good. Regarding the overall quality assessment, four studies had good overall ratings^10,11,14,15^. On the other hand, two studies had fair overall ratings^12,13^. One study had a participant eligibility rate of <50%^13^. In the Meo et al.^12^ study, the timeframe was not sufficient to reasonably see an association between exposure and outcome. Moreover, the e-cigarette was not assessed more than once over time, and all the subjects selected were not from the same or similar populations. In the Campagna et al.^10^ study, the overall dropout rate was >20%, and the sample size was not large enough to detect a significant difference in the main outcome between groups with at least 80% power^10^. In Figure 2, the domain-level judgments of individual studies using the NIH cross-sectional and cohort quality assessment tools are reported. In Sompa et al.^14^, a cross-sectional clinical study rated as Good quality using the NIH assessment tool, participants were matched by age and BMI with clearly defined inclusion criteria and validated FeNO measurement methods. However, the variance for the reported median FeNO values was not provided, and the small sample size without follow-up limited the strength of causal inference.

**Figure 2:**
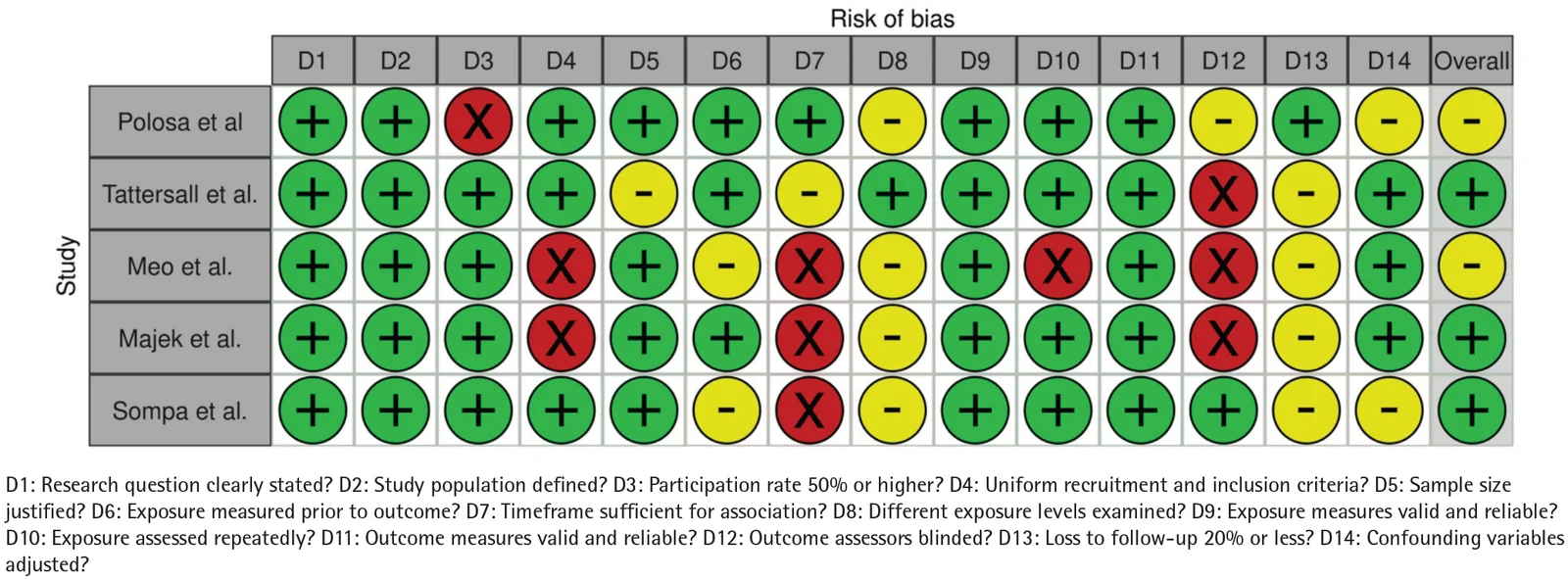
Risk of bias assessment for the observational studies using the NIH quality assessment tool for cross-sectional and cohort studies

### Effects of e-cigarette use on FeNO test

In Meo et al.^12^, 60 healthy males were included in the study, of whom 30 (50%) were daily e-cigarette users with a mean age of 27.07 ± 6.00 years, and 30 (50%) were control subjects who did not consume any tobacco products with a mean age of 25.90 ± 7.72 years. The 30 e-cigarette smokers group only used e-cigarettes and had never used conventional cigarettes. All participants were instructed not to eat or drink any kind of hot or cold beverage for at least 2 hours before testing. They were also asked not to use their e-cigarette device for at least one hour before testing^12^. The reported FeNO in the e-cigarette users’ group was 18 ± 11.05 ppb, whereas the control group was 21.67 ± 8.58 ppb; however, there was nostatistical difference between the groups (p=0.16).

The Tattersall et al.^15^ study included 395 individuals who were divided into three groups based on smoking behavior. Group 1 included 164 (41.5%) exclusive e-cigarette users, of whom 64 (39%) were female and 100 (61%) were male, with a mean age of 27.4 ± 10.6 years for both females and males. Group 2 included 117 (29.6%) participants who smoked conventional cigarettes exclusively, of whom 52 (44.4%) were female and 65 (55.6%) were male, with a mean age of 42.8 ± 13.8 years for both females and males. Group 3 included 114 (28.8%) control participants, of which 57 (50%) were female and 57 (50%) were male, with a mean age of 30.8 ± 11.9 years for both females and males. FeNO was measured using the online breath condensate method via a rapid-response chemiluminescent analyzer. The mean FeNO reported for group 1 users was 14.5 ± 14.7 ppb, whereas for group 2 it was 9.7 ± 12.3 ppb, and for group 3 it was 16.2 ± 11.0 ppb. Group 1 demonstrated lower values than Group 3 and was statistically significant (p=0.013).

In a prospective longitudinal study by Polosa et al.^13^, 21 adult participants were recruited. A total of 9 (43%) were daily e-cigarette users (6 male and 3 female), with a mean age of 29.7 ± 6.1 years, who had never used conventional cigarettes. A group of 12 (57%) participants (8 male and 4 female), with a mean age of 32.5 ± 7.0 years, of non-smokers were included as controls. Six e-cigarette users consumed lower strength nicotine-containing e-liquid, while three consumed zero-nicotine strength e-liquid. Controls were those who had never smoked or had fewer than 100 cigarettes in their lifetime. For 3.5 years, annual visits were planned. Before every visit, participants were advised to stop vaping for at least 60 minutes. The baseline median FeNO for e-cigarettes was 21.1 ppb (IQR: 16.2–24.5), whereas the baseline median FeNO for the control group was 18.6 ppb (IQR: 17.6–25.7). After 60 minutes from the exposure, the median FeNO level for the e-cigarette group was 20.0 ppb (IQR: 18.2–22.7), and the median FeNO level for the control group was 20.0 ppb (IQR: 16.2–23.4). Upon investigation, no significant differences were observed between the two groups (p=0.89).

Majek et al.^11^ published a study in 2023 that included 160 healthy adults, of which 40 (25%) were e-cigarette users (20 males and 20 females), 40 (25%) were non-smokers (19 males and 21 females) as control groups, 40 (25%) were heated tobacco product users, and 40 (25%) conventional cigarette smokers (18 males and 22 females). The median age for the e-cigarette users’ group was 21 years (IQR: 20–22), 25.5 years (IQR: 22–28) for the control group, and 23 years (IQR: 21–24.5) for the conventional cigarette smokers’ group. The baseline mean FeNO for the e-cigarette’s users’ group was higher than the control group, 16.9 ± 6.5 versus 13.2 ± 5.9 ppb, which was statistically significant (p=0.01). And the baseline mean FeNO for the conventional cigarette smokers was 10.3 ± 5.7 ppb, which was statistically significant compared to the control group (p=0.04). Also, there was a difference in the baseline FeNO level between conventional cigarette smokers and e-cigarette group (p<0.01). This study’s limitations include the fact that there are differences in smoking amounts and daily use among participants. Participants were selected depending on their current usage regarding given type of product, and their prior smoking might affect their present status.

In the Campagna et al.^10^ study, a total of 300 ‘healthy’ smokers were initially recruited, 190 (63.33%) were male and 110 (36.67%) were female with a mean age of 44.0 ± 12.5 years. The study was designed to assess how e-cigarettes reduce the consumption of conventional cigarettes. Only 134 completed the study and participants were randomized into three study arms to receive e-cigarette kits containing either 2.4% nicotine (A), 1.8% nicotine (B), or no nicotine (C) using a computer-generated randomization sequence. Group A (n=49) who received (12 weeks of ‘Original 2.4%’), Group B (n=45) who received (6 weeks of ‘Original 2.4%’) and a further (6 weeks of ‘Category 1.8%’), and Group C (n=40) who received (12 weeks of ‘Original 0%’). In the author’s analysis, participants were grouped as either quitters (those who completely stopped smoking), reducers, or those who failed to quit. FeNO levels were measured at baseline and throughout the year. The results showed that quitters had significantly increased levels of FeNO over time and reached nearly normal levels by the end of the year. Conversely, no significant differences in FeNO levels were seen in participants who failed to quit or reduced their consumption of cigarettes. At baseline, the median FeNO was 6.6 ppb (IQR: 4.3–8.4) for failures, 5.9 ppb (IQR: 5.0–7.8) for reducers, and 5.5 ppb (IQR: 4.5–6.9) for quitters. At week 52 it was 7.0 ppb (IQR: 5.5–9.9), 7.9 ppb (IQR: 6.0–10.8), and 17.7 ppb (IQR: 13.3–18.9).

In the Sompa et al.^14^ study, 82 participants were included, consisting of 22 healthy non-smokers, 20 exclusive e-cigarette users, 20 conventional cigarette smokers, and 20 dual users who used both products. Participants were matched by age and body mass index (BMI), with a median age ranging between 26 and 30 years across all groups. All participants had normal lung function, confirmed by an FEV1/FVC ratio ≥0.7, and none had a history of allergies, lung disease, or recent infections. The e-cigarette users reported a median duration of use of 2.7 years (IQR: 2–3). FeNO was measured using the NIOX VERO device following ATS/ERS guidelines. The median FeNO level was significantly higher in e-cigarette users (14 ppb, p=0.04) compared to healthy non-smokers (11 ppb), while cigarette smokers exhibited significantly lower FeNO levels (9 ppb, p=0.04). Dual users showed a similar trend with higher FeNO compared to cigarette smokers (p=0.03). The study also found that e-cigarette and dual users demonstrated increased bronchial hyperresponsiveness and elevated oxidative stress markers, indicating early local and systemic inflammatory changes even after only 1–3 years of use.

In Figure 3, the FeNO levels of e-cigarette smokers, controls, and conventional cigarette smokers are plotted.

**Figure 3:**
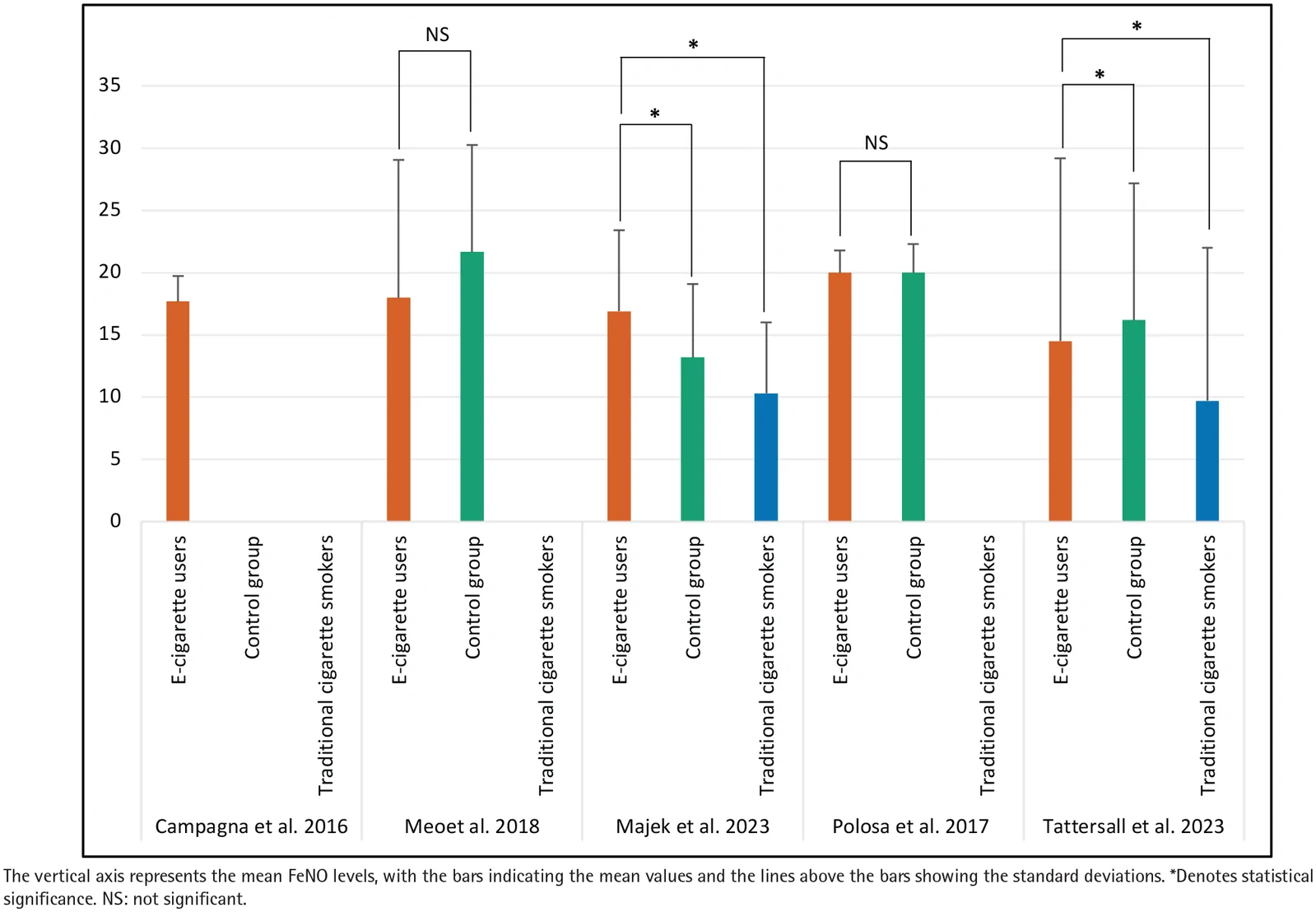
FeNO levels among e-cigarette users, control groups, and traditional cigarette smokers in 5 observational studies

### Data synthesis

Overall, three studies were examined, with 234 participants in the e-cigarette users groups and 184 in the control groups. There was no significant difference between the two groups. The overall mean difference was -0.26 (95% CI: -9.74–9.22). There was considerable heterogeneity indicating inconsistent effects in size and/or direction. The I2 indicated heterogeneity, with 80% of the variability between studies. In Figure 4, a forest plot of the three included studies is illustrated.

**Figure 4:**
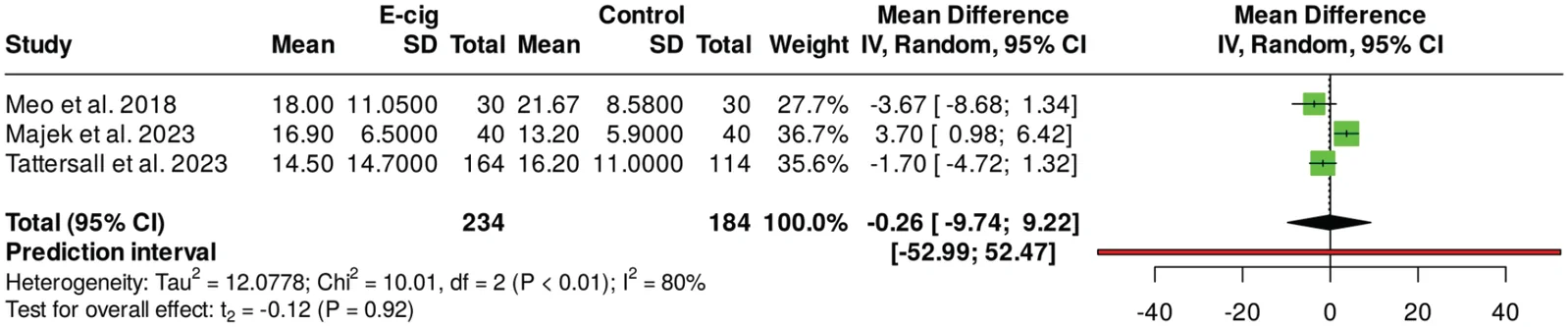
Forest plot of the metanalysis comparing mean FeNO differences between e-cigarette users and controls

## DISCUSSION

A total of 792 participants were included in this systematic review, of whom 397 were long-term e-cigarette users. This review was designed to evaluate the impact of e-cigarette usage on airway inflammation via FeNO measurements. Overall, as determined by the studies that were reviewed, the results were heterogeneous and inconclusive. The difference in the values of different studies showed variations, as some studies reported that e-cigarette users had higher FeNO levels compared to other control subjects, while others found lower levels. Sompa et al.^14^ additionally reported significantly higher median FeNO levels in e-cigarette users compared to healthy non-smokers and lower levels in conventional cigarette smokers, though the variance for the median FeNO values was not reported. The meta-analysis of the included studies revealed no difference in FeNO level between long-term e-cigarette smokers and control participants.

FeNO is frequently used as a marker of airway inflammation driven by eosinophils, but its accuracy depends on additional variables. It performs best when interpreted alongside other clinical factors. A decrease in FeNO levels can result from various mechanisms, including smooth muscle activity, altered mucus secretion, impaired nitric oxide transfer from the mucosa to the airway lumen, or interactions with inhaled substances that promote oxidation^16^. In previous studies, there had been conflicting findings in comparing FeNO levels before and after cigarette smoking^17,18^. The lack of consistency between studies could be attributed to the different post-exposure collection times; studies that measured FeNO immediately or 1 minute after e-cigarette exposure reported decreased FeNO levels^19,20^. On the other hand, when FeNO was examined after a longer period of time, such as 15 minutes to 2 hours, there was either no change or a level increase^17,20^.

When evaluating the effect of conventional cigarettes on FeNO levels, a previous study found that FeNO levels increase after acute conventional cigarette smoke exposure^21^. Some studies had shown that prolonged exposure to cigarette smoke reduced FeNO levels^18^. Nonetheless, Balint et al.^22^ did not find any substantial short term increase in the eNO amounts after two cigarette smokers. Conversely, Habib et al.^23^ showed that the level of exhaled FeNO was lower in young adult smokers than in non-smokers. However, no relationship of FeNO was noticed with the number or length of time cigarettes were smoked per day^24^.

Despite the heterogeneity of the results, there might be some preliminary evidence that long-term use of e-cigarettes requires more research, particularly regarding the severe healthcare implications it has for young individuals. The FeNO test is known to be sensitive to eosinophilic inflammatory reactions^25^; however, neutrophils have an essential role in inflammation initiation and resolution, they rapidly recruited to sites of inflammation and tissue injury. After exposure to e-cigarettes, neutrophils were unable to function properly and changed their phenotype, which caused significant dysfunction in neutrophils^25^.

### Limitations

Several limitations were observed during this systematic review and meta-analysis. First, the dose and frequency of e-cigarette use could not be calculated because of the differences in depth of inhalation and frequency. Consensus on how e-cigarette use should be implemented to adequately assess the effects of its use. In addition, the included research studies did not address the variations in e-cigarette types, nicotine strengths, flavors, and chemical additives, despite the significant effect these factors have on study outcomes. According to Tillery et al.^26^, users of different e-cigarette devices, such as POD systems and MODs, experience distinct chemical exposures and exhibit differing usage patterns. Second, there was a high variation between studies on the duration of e-cigarette use. Third, there were a limited number of studies included in this review, and the sample size was relatively small. Fourth, the majority of participants were male, which limits the applicability of the findings to female populations. Fifth, residual confounding may affect the results, as various potential confounders, such as allergic rhinitis, eosinophilic bronchitis, or atopic dermatitis, can increase FeNO levels^4^. Physical activity, another factor known to increase FeNO levels, was also not accounted for^4^. Moreover, while diseases such as asthma, COPD, and cystic fibrosis were excluded, the lack of reporting on other potential confounders limits the interpretation of results. Sixth, it is important to note that two of the included studies were sponsored by the e-cigarette related industry^10,13^, which may introduce bias and influence the reported outcomes. Lastly, most of the included studies were cross-sectional in design, which makes it difficult to prove the causal relationship between e-cigarette use and FeNO levels.

### Future research

FeNO may not be useful as a tool to measure the long-term impact of e-cigarettes on human participants due to its sensitivity to eosinophilic airway inflammation; however, further research is needed to validate its efficacy for evaluating the effect of e-cigarettes on long-term e-cigarette use. For this reason, longitudinal studies should involve participants using diverse mediums, including FeNO, because the impact of NO is not clear among e-cigarette users. Future work might consider including the diffusing capacity of the lungs for carbon monoxide (DLCO). In the Muise et al.^27^ preliminary report of 65 youth, they pointed out DLCO and 6-minute walk test (6MWT) abnormalities and emphasized the need for full pulmonary function testing (PFTs) in e-cigarette users’ assessments. However, more investigations are required to understand the effects of e-cigarettes on lung health by employing markers that are particular to other white blood cells, notably neutrophils. In addition, more research is needed to investigate the effects of different types and patterns of e-cigarette use on FeNO levels and other pulmonary parameters.

## CONCLUSIONS

The results across studies were heterogeneous and inconclusive, highlighting the variability in findings related to the impact of long-term e-cigarette use on airway inflammation. These inconsistencies underscore the need for further research, particularly well-powered longitudinal prospective studies, to better understand the long-term effects of e-cigarette use on inflammatory biomarkers such as FeNO.

## Supplementary Material



## Data Availability

Data sharing is not applicable to this article as no new data were created.
