# Supplementary material for: The effect of long-term e-cigarette use on the inflammation marker fractional exhaled nitric oxide (FeNO): A systematic review and meta-analysis
Source: Tob Induc Dis. 2026 Sep 30;24:10.18332/tid/224361. doi: 10.18332/tid/224361 (PMC13629185; doi:10.18332/tid/224361)
Supplement: Supplementary file 1 [file TID-24-179-s1.pdf]

Tobacco Induced Diseases

Supplementary file

© 2026 Almeshari M.A.
et al.

DOI:
10.18332/tid/224361

The content has been provided by the author(s) and has not been reviewed, verified, or endorsed by European Publishing. It may not have undergone peer review. The views, opinions, and recommendations expressed are solely those of the author(s) and do not necessarily reflect the position of European Publishing. European Publishing accepts no responsibility or liability for any consequences arising from the use of, or reliance on, this content.

Appendix A. PRISMA 2020 Checklist

Section and Topic	Item #	Checklist item	Location where item is reported
**TITLE**			
Title	1	Identify the report as a systematic review.	1
**ABSTRACT**			
Abstract	2	See the PRISMA 2020 for Abstracts checklist.	2
**INTRODUCTION**			
Rationale	3	Describe the rationale for the review in the context of existing knowledge.	3
Objectives	4	Provide an explicit statement of the objective(s) or question(s) the review addresses.	3
**METHODS**			
Eligibility criteria	5	Specify the inclusion and exclusion criteria for the review and how studies were grouped for the syntheses.	3-4
Information sources	6	Specify all databases, registers, websites, organisations, reference lists and other sources searched or consulted to identify studies. Specify the date when each source was last searched or consulted.	3
Search strategy	7	Present the full search strategies for all databases, registers and websites, including any filters and limits used.	4
Selection process	8	Specify the methods used to decide whether a study met the inclusion criteria of the review, including how many reviewers screened each record and each report retrieved, whether they worked independently, and if applicable, details of automation tools used in the process.	4
Data collection process	9	Specify the methods used to collect data from reports, including how many reviewers collected data from each report, whether they worked independently, any processes for obtaining or confirming data from study investigators, and if applicable, details of automation tools used in the process.	4
Data items	10a	List and define all outcomes for which data were sought. Specify whether all results that were compatible with each outcome domain in each study were sought (e.g. for all measures, time points, analyses), and if not, the methods used to decide which results to collect.	4
	10b	List and define all other variables for which data were sought (e.g. participant and intervention characteristics, funding sources). Describe any assumptions made about any missing or unclear information.	4
Study risk of bias assessment	11	Specify the methods used to assess risk of bias in the included studies, including details of the tool(s) used, how many reviewers assessed each study and whether they worked independently, and if applicable, details of automation tools used in the process.	5
Effect measures	12	Specify for each outcome the effect measure(s) (e.g. risk ratio, mean difference) used in the synthesis or presentation of results.	5
Synthesis methods	13a	Describe the processes used to decide which studies were eligible for each synthesis (e.g. tabulating the study intervention characteristics and comparing against the planned groups for each synthesis (item #5)).	5
	13b	Describe any methods required to prepare the data for presentation or synthesis, such as handling of missing summary statistics, or data conversions.	5
	13c	Describe any methods used to tabulate or visually display results of individual studies and syntheses.	5
	13d	Describe any methods used to synthesize results and provide a rationale for the choice(s). If meta-analysis was performed, describe the model(s), method(s) to identify the presence and extent of statistical heterogeneity, and software package(s) used.	5
	13e	Describe any methods used to explore possible causes of heterogeneity among study results (e.g. subgroup analysis, meta-regression).	5
	13f	Describe any sensitivity analyses conducted to assess robustness of the synthesized results.	-
Reporting bias assessment	14	Describe any methods used to assess risk of bias due to missing results in a synthesis (arising from reporting biases).	5
Certainty assessment	15	Describe any methods used to assess certainty (or confidence) in the body of evidence for an outcome.	5

Appendix A. PRISMA 2020 Checklist

Section and Topic	Item #	Checklist item	Location where item is reported
**RESULTS**			
Study selection	16a	Describe the results of the search and selection process, from the number of records identified in the search to the number of studies included in the review, ideally using a flow diagram.	5
	16b	Cite studies that might appear to meet the inclusion criteria, but which were excluded, and explain why they were excluded.	5
Study characteristics	17	Cite each included study and present its characteristics.	5-6
Risk of bias in studies	18	Present assessments of risk of bias for each included study.	6
Results of individual studies	19	For all outcomes, present, for each study: (a) summary statistics for each group (where appropriate) and (b) an effect estimate and its precision (e.g. confidence/credible interval), ideally using structured tables or plots.	6-8
Results of syntheses	20a	For each synthesis, briefly summarise the characteristics and risk of bias among contributing studies.	8
	20b	Present results of all statistical syntheses conducted. If meta-analysis was done, present for each the summary estimate and its precision (e.g. confidence/credible interval) and measures of statistical heterogeneity. If comparing groups, describe the direction of the effect.	8
	20c	Present results of all investigations of possible causes of heterogeneity among study results.	8
	20d	Present results of all sensitivity analyses conducted to assess the robustness of the synthesized results.	-
Reporting biases	21	Present assessments of risk of bias due to missing results (arising from reporting biases) for each synthesis assessed.	5
Certainty of evidence	22	Present assessments of certainty (or confidence) in the body of evidence for each outcome assessed.	9
**DISCUSSION**			
Discussion	23a	Provide a general interpretation of the results in the context of other evidence.	9
	23b	Discuss any limitations of the evidence included in the review.	9-10
	23c	Discuss any limitations of the review processes used.	10
	23d	Discuss implications of the results for practice, policy, and future research.	10
**OTHER INFORMATION**			
Registration and protocol	24a	Provide registration information for the review, including register name and registration number, or state that the review was not registered.	3
	24b	Indicate where the review protocol can be accessed, or state that a protocol was not prepared.	3
	24c	Describe and explain any amendments to information provided at registration or in the protocol.	-
Support	25	Describe sources of financial or non-financial support for the review, and the role of the funders or sponsors in the review.	-
Competing interests	26	Declare any competing interests of review authors.	11
Availability of data, code and other materials	27	Report which of the following are publicly available and where they can be found: template data collection forms; data extracted from included studies; data used for all analyses; analytic code; any other materials used in the review.	-

From: Page MJ, McKenzie JE, Bossuyt PM, Boutron I, Hoffmann TC, Mulrow CD, et al. The PRISMA 2020 statement: an updated guideline for reporting systematic reviews. BMJ 2021;372:n71. doi: 10.1136/bmj.n71

Appendix B. The Search terms

Population	Exposure	Comparator	Outcome
Adult*.ti,ab.	E Cigarette Vapor.ti,ab.		Exhaled Nitric Oxide Test.ti,ab.
Aged.ti,ab.	E-Cigarette*.ti,ab.		FeNO.ti,ab.
Aged, 80 and over.ti,ab.	Electronic Cigarette Vapor.ti,ab.		FeNO test*.ti,ab.
Frail Elderly.ti,ab.	Cigarette Vapor, Electronic.ti,ab.		Fractional Exhaled Nitric Oxide.ti,ab.
Middle Aged.ti,ab.	Vapor, Electronic Cigarette.ti,ab.		Innate Inflammatory Response*.ti,ab.
Young Adult*.ti,ab.	E-Cig Use*.ti,ab.		Acute-Phase Reaction.ti,ab.
Smoker*.ti,ab.	E Cig Use.ti,ab.		
Vaper*.ti,ab.	Use, E-Cig.ti,ab.		
e-cigarettes smoker*.ti,ab.	ECig*.ti,ab.		
e-cig smoker*.ti,ab.	Vape*.ti,ab.		
	E-Cigarette Use*.ti,ab.		
	E Cigarette Use.ti,ab.		
	Use, E-cigarette.ti,ab.		
	Nicotine Vaping*.ti,ab.		
	Vaping*, Nicotine.ti,ab.		
	Ecigarette Use*.ti,ab.		
	Use*, Ecigarette.ti,ab.		
	Electronic Cigarette Use*.ti,ab.		
	Cigarette Use, Electronic.ti,ab.		
	Use, Electronic Cigarette.ti,ab.		
	Electronic Nicotine Delivery System.ti,ab.		
	Electronic Cigarette*.ti,ab.		
	E-Cig*.ti,ab.		
	E Cig*.ti,ab.		
	E Cigarette*.ti,ab.		
	E-Cigarette*.ti,ab.		
	Electronic Cigarette.ti,ab.		
	Cigarette*, Electronic.ti,ab.		

Appendix C. The Searching Query

1	Adult*.ti,ab.
2	Aged.ti,ab.
3	Middle Aged.ti,ab.
4	Smoker*.ti,ab.
5	Vaper*.ti,ab.
6	Frail Elderly.ti,ab.
7	e-cigarettes smoker*.ti,ab.
8	e-cig smoker*.ti,ab.
9	E Cigarette Vapor.ti,ab.
10	E-Cigarette*.ti,ab.
11	Electronic Cigarette Vapor.ti,ab.
12	E-Cig Use*.ti,ab.
13	ECig*.ti,ab.
14	Vape*.ti,ab.
15	E-Cigarette Use*.ti,ab.
16	Nicotine Vaping*.ti,ab.
17	Ecigarette Use*.ti,ab.
18	Electronic Cigarette Use*.ti,ab.
19	Electronic Nicotine Delivery System.ti,ab.
20	Electronic Cigarette*.ti,ab.
21	E-Cig*.ti,ab.
22	E Cig*.ti,ab.
23	E Cigarette*.ti,ab.
24	E-Cigarette*.ti,ab.
25	Electronic Cigarette.ti,ab.
26	ecig*.ti,ab.
27	vapor*.ti,ab.
28	aerosol*.ti,ab.
29	e-juice*.ti,ab.
30	e-liquid*.ti,ab.
31	vap*.ti,ab.
32	Electronic-cigar*.ti,ab.
33	e-cigar*.ti,ab.
34	smoking-alternative*.ti,ab.
35	electronic-hookah*.ti,ab.
36	e-hookah*.ti,ab.
37	e-nicotine.ti,ab.
38	Exhaled Nitric Oxide Test.ti,ab.
39	FeNO.ti,ab.
40	FeNO test*.ti,ab.
41	Fractional Exhaled Nitric Oxide.ti,ab.
42	Innate Inflammatory Response*.ti,ab.
43	eNO.ti,ab.
44	nitric oxide.ti,ab.
45	Acute-Phase Reaction.ti,ab.
46	Exhaled Nitric Oxide.ti,ab.
47	38 or 39 or 40 or 41 or 42 or 43 or 44 or 45 or 46

48	9 or 10 or 11 or 12 or 13 or 14 or 15 or 16 or 17 or 18 or 19 or 20 or 21 or 22 or 23 or 24 or 25 or 26 or 27 or 28 or 29 or 30 or 31 or 32 or 33 or 34 or 35 or 36 or 37
49	1 or 2 or 3 or 4 or 5 or 6 or 7 or 8
50	47 and 48 and 49

Embase 1974 to 2024 February 13

Ovid MEDLINE(R) ALL 1946 to February 13, 2024

Appendix D. MOOSE Checklist for Meta-analyses of Observational Studies

Item No	Recommendation	Reported on Page No
Reporting of background should include		
1	Problem definition	3
2	Hypothesis statement	3
3	Description of study outcome(s)	3
4	Type of exposure or intervention used	3
5	Type of study designs used	3
6	Study population	3
Reporting of search strategy should include		
7	Qualifications of searchers (eg, librarians and investigators)	-
8	Search strategy, including time period included in the synthesis and key words	3
9	Effort to include all available studies, including contact with authors	3
10	Databases and registries searched	3
11	Search software used, name and version, including special features used (eg, explosion)	4
12	Use of hand searching (eg, reference lists of obtained articles)	-
13	List of citations located and those excluded, including justification	5
14	Method of addressing articles published in languages other than English	-
15	Method of handling abstracts and unpublished studies	5
16	Description of any contact with authors	-
Reporting of methods should include		
17	Description of relevance or appropriateness of studies assembled for assessing the hypothesis to be tested	3
18	Rationale for the selection and coding of data (eg, sound clinical principles or convenience)	4
19	Documentation of how data were classified and coded (eg, multiple raters, blinding and interrater reliability)	4
20	Assessment of confounding (eg, comparability of cases and controls in studies where appropriate)	4
21	Assessment of study quality, including blinding of quality assessors, stratification or regression on possible predictors of study results	5
22	Assessment of heterogeneity	5
23	Description of statistical methods (eg, complete description of fixed or random effects models, justification of whether the chosen models account for predictors of study results, dose-response models, or cumulative meta-analysis) in sufficient detail to be replicated	5
24	Provision of appropriate tables and graphics	6
Reporting of results should include		
25	Graphic summarizing individual study estimates and overall estimate	8
26	Table giving descriptive information for each study included	6
27	Results of sensitivity testing (eg, subgroup analysis)	-
28	Indication of statistical uncertainty of findings	8

Item No	Recommendation	Reported on Page No
Reporting of discussion should include		
29	Quantitative assessment of bias (eg, publication bias)	5
30	Justification for exclusion (eg, exclusion of non-English language citations)	5
31	Assessment of quality of included studies	6
Reporting of conclusions should include		
32	Consideration of alternative explanations for observed results	9
33	Generalization of the conclusions (ie, appropriate for the data presented and within the domain of the literature review)	10
34	Guidelines for future research	10
35	Disclosure of funding source	11

From: Stroup DF, Berlin JA, Morton SC, et al, for the Meta-analysis Of Observational Studies in Epidemiology (MOOSE) Group. Meta-analysis of Observational Studies in Epidemiology. A Proposal for Reporting. *JAMA*. 2000;283(15):2008-2012. doi: 10.1001/jama.283.15.2008.
